# KGBN: Augmenting and optimizing logical gene regulatory networks using knowledge graphs

**DOI:** 10.64898/2026.01.29.702644

**Published:** 2026-01-30

**Authors:** Luna Xingyu Li, Yue Zhang, Boris Aguilar, Tazein Shah, John H Gennari, Guangrong Qin

**Affiliations:** 1 Institute for Systems Biology, 401 Terry Ave N, 98109, WA, United States; 2 Department of Medical Education and Biomedical Informatics, University of Washington, 1410 NE Campus Pkwy, 98195, WA, United States

**Keywords:** gene regulatory networks, logical models, knowledge graphs, acute myeloid leukemia, systems biology

## Abstract

**Motivation:**

Logical gene regulatory network (GRN) models provide interpretable, mechanistic representations of cellular regulation and are widely used in systems biology. However, most existing models remain incomplete, context-specific, and difficult to extend to comprehensive GRNs, limiting their broader applicability to tasks such as drug-response prediction and precision medicine.

**Results:**

We present KGBN (Knowledge Graph–augmented Boolean Network modeling), a computational workflow for systematically augmenting logical GRN models. KGBN incorporates regulatory interactions derived from curated knowledge graphs as alternative logical rules while preserving the validated structure of existing models. Rule probabilities are optimized against experimental data to represent regulatory uncertainty and achieve data-driven calibration. Applying KGBN to acute myeloid leukemia, we show that extending an existing GRN with drug-target pathways and training against *ex vivo* drug-response data yields mutation-specific models that recapitulate known therapeutic sensitivities and signaling dependencies, demonstrating the utility of KGBN for interpretable, context-aware GRN modeling.

**Availability and implementation:**

KGBN is available at https://github.com/IlyaLab/KGBN.

## 1 Introduction

Systems biology aims to explain how molecular components interact to produce emergent cellular behaviors, disease phenotypes, and treatment responses. Mathematical models play a central role in this effort by translating biological knowledge into executable representations that can be simulated and tested (1; 2). Among them, logical models have become a widely adopted formalism for representing gene regulatory networks (GRNs) by describing the qualitative activation states of genes and proteins and using logical rules to capture their regulatory relationships (3–5).

GRN modeling has been instrumental in revealing the mechanisms of complex diseases. In acute myeloid leukemia (AML), for example, logical models have been used to capture hematopoietic stem cell differentiation and the dysregulation of transcriptional programs driven by oncogenic mutations such as *FLT3*, *NPM1*, and *RUNX1* (6–8). They have been used to study a wide range of biological processes, including signaling crosstalk, drug response, and resistance mechanisms in various diseases (e.g., breast cancer (9) and pancreatic cancer (10)). However, these models are typically constructed for specific biological questions, often omitting drug targets or signaling intermediates, and encode a single deterministic regulatory logic per node (11; 12). These limitations hinder their ability to represent regulatory uncertainty, capture patient heterogeneity, or adapt to new experimental contexts such as drug perturbation screens.

Recent advances provide an opportunity to address these challenges. First, biomedical knowledge graphs systematically curate causal relationships among genes, proteins, drugs, and phenotypes, offering a scalable resource for identifying missing regulatory interactions and drug–target connections (13; 14). Second, probabilistic Boolean networks (PBNs) extend classical Boolean models by allowing multiple alternative regulatory rules per node, thereby explicitly representing uncertainty, context dependence, and competing biological hypotheses (15; 16). Third, large-scale experimental datasets, including ex vivo drug-response and multi-omics profiling, enable data-driven calibration of model parameters, transforming static logic models into quantitatively informed predictive systems.

Here, we introduce KGBN (Knowledge Graph-augmented Boolean Network modeling), a computational framework that integrates these elements into a unified workflow for augmentation and optimization of GRN models. KGBN uses knowledge graphs to augment existing logical models with disease- and drug-relevant regulators, encodes alternative regulatory hypotheses using PBNs, and optimizes rule probabilities against experimental data to improve predictive fidelity. We demonstrate the utility of this framework through drug response prediction in AML and through reproduction of a published pancreatic cancer model, illustrating both its predictive capability and its reliability as a general execution and extension platform for GRN models. With the potential to apply to diverse biological and disease contexts, this framework will facilitate mechanistic hypothesis generation, mutation-specific drug response analysis, and *in silico* exploration of therapeutic combinations.

## 2 Materials and Methods

### 2.1 Boolean networks and PBNs

We model a gene regulatory network as a discrete dynamical system composed of binary state variables. Let $V=\left\{x_{1},\ldots ,x_{n}\right\}$ denote the set of nodes, where each node $x_{i}(t)\in \{0,1\}$ represents the activity state of a gene, protein, or phenotype at discrete time t. In a Boolean network, the state of each node is updated by a Boolean function $x_{i}(t+1)=f_{i}\left(x_{1}(t),\ldots ,x_{n}(t)\right)$, where $f_{i}:\{0,1{\}}^{n}\to \{0,1\}$ encodes the regulatory logic of node i. The global network state is given by $x(t)=\left(x_{1}(t),\ldots ,x_{n}(t)\right)$. In KGBN, we adopt a synchronous update scheme, where all node states are updated simultaneously at each discrete time step.

PBNs generalize this formulation by allowing multiple alternative update rules per node. For each node $x_{i}$, we define a set of candidate Boolean functions ${ℱ}_{i}=\left\{f_{i1},\ldots ,f_{iK_{i}}\right\}$ together with an associated probability vector $p_{i}=\left(p_{i1},\ldots ,p_{iK_{i}}\right),\sum_{j=1}^{K_{i}}p_{ij}=1$. At each update step, one function $f_{ij}$ is selected according to $p_{i}$, and the network state is updated accordingly. This stochastic rule selection induces a Markov chain over the global state space $\{0,1{\}}^{n}$.

In KGBN, this formulation provides a natural mechanism for incorporating regulatory information derived from knowledge graphs. The original Boolean rule of a node is preserved as one element of ${ℱ}_{i}$, while additional rules derived from knowledge-graph interactions are introduced as alternative elements of ${ℱ}_{i}$. The probability vector $p_{i}$ therefore controls the relative contribution of competing regulatory hypotheses.

The steady-state distribution π is defined as the invariant distribution of the Markov chain induced by the PBN, satisfying π=πP, where P is the state transition matrix determined by probabilistic rule selection (17). Steady states in KGBN are estimated using either Monte Carlo simulation, by sampling long stochastic trajectories, or a two-state Markov chain approximation for ergodic PBNs, enabling efficient evaluation of model outputs during rule-probability optimization (15).

### 2.2 The KGBN workflow

**Fig. 1.**
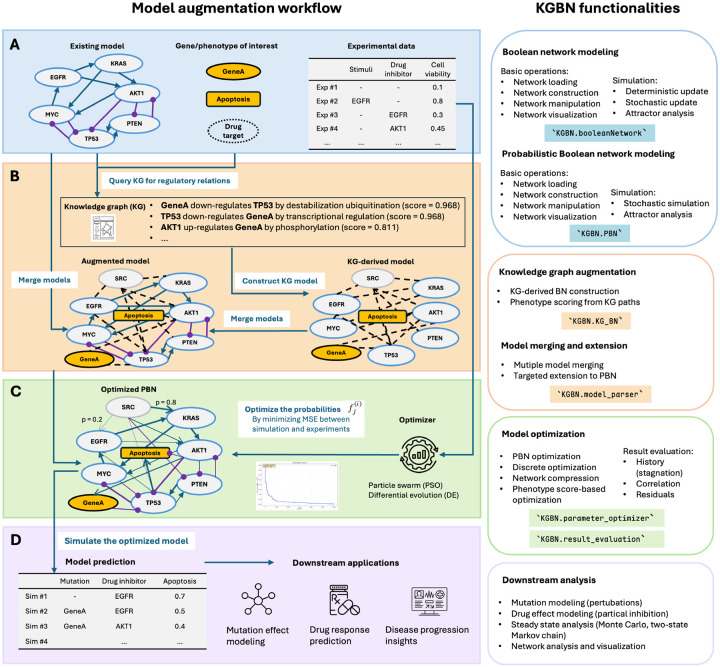
Overview of the workflow and KGBN core functionalities. (A) Identifying and standardizing existing models, genes, phenotypes, and experimental data. (B) Extending the model via knowledge graph (KG) and probabilistic Boolean networks (PBNs). (C) Optimizing the PBN using experimental data. (D) Simulating and evaluating the optimized model.

KGBN is designed as a modular workflow that augments existing logical gene regulatory network models with knowledge-driven structure and data-driven calibration. As illustrated in Figure 1, the workflow proceeds from standardized model and data preparation (A), through systematic network augmentation using curated causal knowledge (B), to probabilistic model optimization (C) and downstream simulation (D). Users can adapt the workflow to different disease contexts, data types, and modeling objectives. The following subsections describe each step of the workflow in detail.

#### 2.2.1 Identifying and standardizing models

The workflow starts with collecting existing models based on the biological questions. We identify additional genes and phenotypes of interest that were not included in the model, including frequent mutated genes, key regulators of disease, drug targets, and phenotypes linked to experimental observations.

Conventional names are often used in models, e.g., *RAS*, a gene family involved in cell growth and cancer development that includes *KRAS* and *NRAS*. To facilitate knowledge graph lookup, all entities in the network should be normalized to their standard formats. For genes, KGBN accepts either standard names/symbols in HGNC (for human models) and NCBI Gene (for other species), or gene IDs in NCBI; for proteins, it accepts their IDs or names as in UniProt.

#### 2.2.2 Extending the model via knowledge graphs

**Fig. 2.**
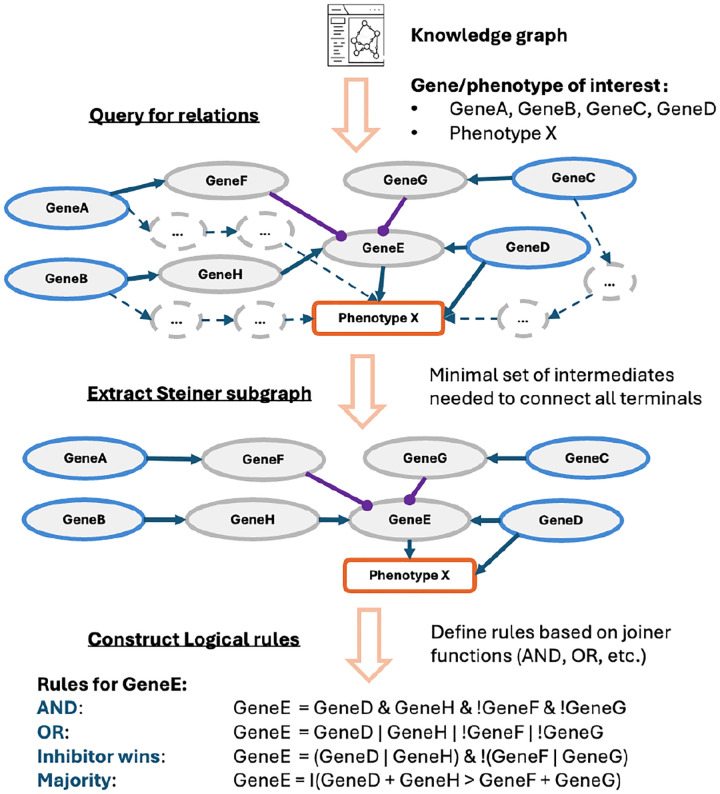
KG–based model extension in KGBN. Query genes (blue-colored) and phenotype (orange) of interest are connected via mediators (gray) based on their regulatory relations from the KG. Steiner subgraph extraction retains the minimal set of intermediate nodes and signed causal edges by removing extra ones (dashed). The resulting subgraph is converted into Boolean rules using configurable joiner functions. Example rules are shown for GeneE, the important intermediate node identified from the KG.

To augment existing GRN models, we develop a pipeline to query the SIGNOR database (the SIGnaling Network Open Resource) (18) and construct Boolean network models from curated molecular interactions (Figure 2). Given a query list of genes or proteins, we identify their interactions by computing the Steiner subgraph (19). This minimal connected subgraph contains all query nodes and their shortest paths through the knowledge graph. Within the extracted subgraph, we classify each directed edge as activating or inhibiting. We then apply a configurable joiner function to generate Boolean update rules:

- OR: the node is active if at least one activator is active.
- AND: the node is active only if all activators are active.
- Inhibitor wins: if any inhibitor is active, the target is inactive regardless of the activator state.
- Majority(Plurality): if the number of active activators is greater than (or equal to) the number of active inhibitors, then the target is active.

The knowledge graph query process includes both the genes present in the original model and additional genes of interest, allowing us to retrieve both the direct interactions among the new components and their connections to the pre-existing model. The resulting network captures a broader range of molecular events that influence phenotypes and enables simulations that more accurately reflect mutation–drug response relationships and uncover regulatory dependencies that may represent novel therapeutic targets.

To help quantify the impact of the network on phenotypes, we leverage the ProxPath framework (20). ProxPath quantifies how close genes are to a phenotype by filtering significantly short paths based on Z-score distribution of all relevant paths in SIGNOR. We take the resulting significant relationships and compute a phenotype score directly from simulated node states: regulators whose net path sign is positive are added, while regulators with a net negative sign are subtracted. This yields a quantitative and interpretable signed sum for each phenotype, ready for connecting network simulations with experimental data in the next step.

#### 2.2.3 Probabilistic Boolean Networks extension

We consider two complementary strategies for incorporating new regulatory information derived from knowledge graphs into existing logical models. One strategy fully merges the base model with a knowledge graph–derived network, combining overlapping and unique nodes and regulatory rules into a single, expanded Boolean network. This approach, implemented in our previously described LM-Merger framework, produces a unified model with increased coverage and integrated logic across components (21). In this work, we focus on a more targeted extension strategy based on PBNs, where alternative regulatory rules derived from knowledge graphs are introduced, while the validated structure of the original model is still preserved.

Let the base model be a Boolean network defined on nodes $V=x_{1},\ldots ,x_{n}$, where each node $x_{i}$ is associated with a Boolean update function $f_{i}^{\left(0\right)}$. For each node $x_{i}$ affected by model extension, we construct a set of alternative Boolean rules ${ℱ}_{i}=f_{i}^{\left(0\right)},f_{i}^{\left(1\right)},\ldots ,f_{i}^{\left(K_{i}\right)}$, where $f_{i}^{\left(0\right)}$ is the original rule from the base model and $f_{i}^{\left(k\right)},k\geq 1$, are additional rules derived from knowledge-graph interactions. Each rule set ${ℱ}_{i}$ is associated with a probability vector $p_{i}=\left(p_{i}^{\left(0\right)},p_{i}^{\left(1\right)},\ldots ,p_{i}^{\left(K_{i}\right)}\right)$, subject to the constraint $\sum k=0^{K_{i}}p_{i}^{\left(k\right)}=1$.

This formulation yields a probabilistic Boolean network in which stochastic selection among alternative rules induces a Markov chain over the global state space $\{0,1{\}}^{n}$. The PBN explicitly represents regulatory uncertainty by allowing multiple competing mechanisms to coexist, with their relative influence controlled by the probability vectors $p_{i}$. This structure provides a principled bridge between curated mechanistic knowledge and data-driven model calibration.

#### 2.2.4 Optimizing the PBN using experimental data

To calibrate the extended PBN against experimental observations, we optimize the rule probability vectors $\left\{p_{i}\right\}$ using quantitative phenotype measurements. Each experimental condition m∈𝒞 is defined by a set of node perturbations (e.g., gene inhibition or activation), their efficacies, and an observed phenotype value $y_{m}$, such as drug response or cell viability. The predicted output for condition m is denoted by ${\overset{ˆ}{y}}_{m}(p)$ and is obtained as a signed linear combination of steady-state node activation probabilities.

The optimization problem is formulated as minimization of the mean squared error between simulated and observed phenotype values across all experimental conditions:

$$\underset{p}{\text{min}}\;ℒ(p)=\frac{1}{\left|𝒞\right|}\sum_{m\in 𝒞}{\left({\overset{ˆ}{y}}_{m}(p)-y_{m}\right)}^{2},$$

subject to $p_{i}^{\left(k\right)}\geq 0$ and $\sum_{k}p_{i}^{\left(k\right)}=1$ for all nodes i.

Because the objective is non-convex and the steady-state distribution depends on p, we employ global optimization strategies, specifically particle swarm optimization and differential evolution (22). Early stopping is applied when improvements in the objective fall below a predefined threshold. Model performance is assessed using prediction–observation correlations, residual analysis, and convergence diagnostics to ensure improved fidelity without overfitting.

#### 2.2.5 Simulation and evaluation

After optimization, the PBN is used for steady-state and perturbation simulations to evaluate model behavior under disease and therapeutic contexts. Genetic mutations, knockouts, and drug treatments are modeled by fixing node states or assigning probabilistic activity levels that reflect partial efficacy. Differences in optimized rule probabilities and simulated phenotypes across conditions are examined to identify regulatory rewiring and pathway dependencies associated with specific mutation backgrounds or treatments.

The optimized model also enables predictive analysis of therapeutic response. Models trained on single-drug perturbation data can be used to simulate drug combinations and to predict relative phenotypic changes across genomic contexts. Model predictions are evaluated by comparison with independent experimental datasets and published evidence. In addition, simulated phenotype scores can be linked to clinical outcomes, such as overall survival, to assess the model’s ability to capture disease progression and treatment response.

### 2.3 Datasets

#### 2.3.1 The Beat AML dataset

To optimize and evaluate the augmented model for AML drug response prediction, the Beat AML dataset was used, which comprises a cumulative cohort of 805 AML patients (942 specimens) (23). WES/targeted sequencing mutation calls, inhibitor AUC values, and clinical summary were retrieved from https://biodev.github.io/BeatAML2/ in December 2025.

#### 2.3.2 The FPMTB dataset

The functional precision medicine tumor board (FPMTB) dataset was used to externally validate the model, which contains ex vivo drug-response and multiomics profiling data for 252 samples from 186 patients with AML (24). Mutation data and drug response DSS scores were retrieved from https://zenodo.org/records/7370747 in December 2025.

#### 2.3.3 Data processing

For both datasets, raw mutation calls and drug-response measurements were processed following the same procedures described in the original publications. Briefly, somatic mutation calls were used to define patient-specific mutation profiles. For drug-response, we focused on six targeted drugs for AML (FDA-approved or clinically investigated): Venetoclax, Entospletinib, Ibrutinib, Trametinib, Selumetinib, and Midostaurin — which represent key inhibitors in AML treatment strategies (reviewed in (25)). For brevity, we only describe our results for the first three drugs; results for the other drugs can be found in the Supplementary materials. For both datasets, we created cohorts where patient response data was available for one of these treatments. Drug-response measurements (AUC for Beat AML and DSS for FPMTB) were normalized using min–max scaling to the [0, 1] range, enabling comparison between experimental responses and simulated phenotypic outputs.

Patients were stratified into mutation-defined subgroups based on the presence or absence of recurrent AML drivers, specifically *FLT3*-ITD, *NPM1*, and *DNMT3A*. These mutation profiles were used to define distinct patient groups for model training, simulation, and downstream analysis. They were mapped to model genes and encoded as fixed node states based on curated gene roles (1 for oncogene and 0 for tumor suppressor genes). Drug-response data were aligned to the corresponding inhibitors and samples, and cohorts were split according to the original Beat AML wave definitions (wave 1+2 for training; wave 3+4 for evaluation). Due to the limited sample size in the FPMTB data, we include only mutation groups with at least five patients to reduce variability in drug response.

## 3 Results

### 3.1 The KGBN toolkit

We developed KGBN, an open-source Python library for Boolean network and probabilistic Boolean network modeling, extension, optimization, and analysis, available at https://github.com/IlyaLab/KGBN. It supports the entire logical model augmentation workflow described in Figure 1 and provides a detailed documentation to facilitate adoption. Its key functionalities include:

1. Network construction and manipulation: Load or build models from SBML-qual, text, or connectivity matrices; merge and extend networks; visualization of networks;
2. Boolean network modeling: Perform deterministic or stochastic updates, steady-state analysis, and trajectory simulation;
3. Probabilistic modeling: Simulate PBNs stochastically, perform steady-state analysis, and visualize the network;
4. Knowledge-graph integration: Derive models from causal relationships between biochemical entities and phenotypes;
5. Optimization and evaluation: Fit model predictions to quantitative experimental data;

To demonstrate the correctness and practical applicability of the toolkit, we evaluated KGBN on a published logical model of pancreatic cancer signaling (26). Using the same model structure and simulation protocol reported in the study, we reproduced the results from the publication, where the effectiveness of drug combinations was predicted based on model simulation (Supplementary Fig. S1). These results confirm KGBN’s functionalities and provide a validated foundation for subsequent analysis on more biological contexts. We then apply the pipeline to other studies as shown in the following section.

### 3.2 A use case: AML drug response prediction

We demonstrated the utility of this workflow on a Boolean model that describes frequent mutations and regulatory interactions in AML to predict clinical outcomes in patients (7). By augmenting this model to include specific drug-target genes via KGBN, we are able to better model AML drug response for specific mutations.

#### 3.2.1 Model augmentation on drug targets

**Fig. 3.**
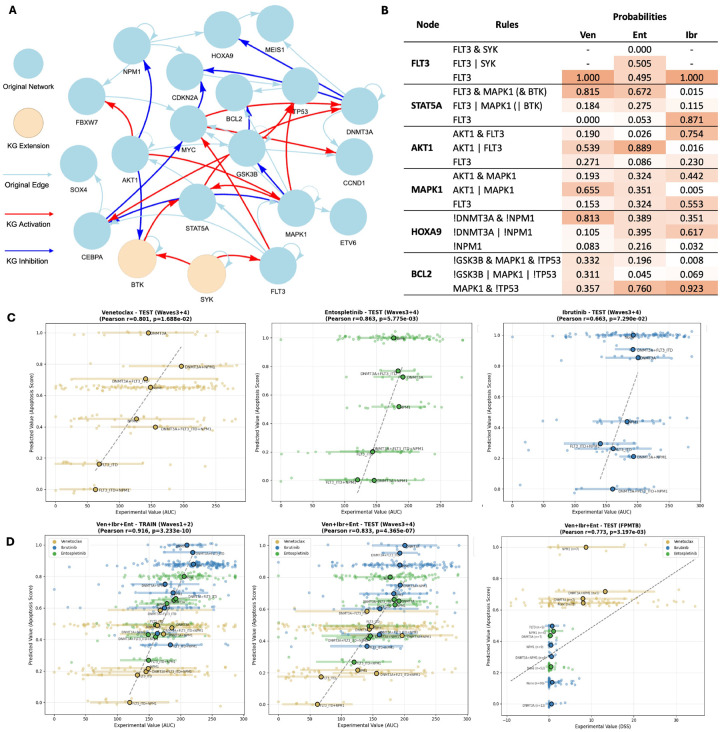
Augmentation and optimization of an AML model on drug response. (A) The extended model. (B) Rule probabilities in optimized PBNs for Venetoclax, Entospletinib, and Ibrutinib, respectively. (C) Correlation of model simulation and experimental measurements in Beat AML test set for models trained on individual drug response data. (D) Correlation of model simulation and experimental measurements for models trained on combined drug response data. For (C) and (D), each dot represents a group of patients with a particular genetic mutation profile. Apoptosis scores were reversed for correlation with AUC values.

Starting with the targeted agents for AML available from the Beat AML dataset, we extended the published AML model to their primary targets and the associated regulators (7). Figure 3A shows the extended model with *SYK* (target of Entospletinib) and *BTK* (target of Ibrutinib), where *BCL2*, target of Venetoclax, is already included in the original model. We queried the SIGNOR knowledge graph for regulatory relationships between these drug targets and existing model genes, and added them as alternative rules in the resulting PBNs. Patient samples were grouped by mutation status of *FLT3*-ITD, *NPM1*, and *DNMT3A*, the three most frequently mutated genes in AML, and the mean drug-response AUC was calculated for each group for training the model (Fig. S2).

To link model simulation to drug efficacy, we derived an ‘apoptosis’ score from the knowledge graph by combining multiple gene statuses as $A=-x_{AKT1}-x_{BCL2}+x_{\text{CDKN}2A}+x_{\text{GSK}3B}-x_{\text{MAPK}1}+x_{\text{MEIS}1}-x_{\text{STAT}5A}+x_{\text{TP}53}$ (see 2.2.2). During optimization, the sign of the apoptosis score was reversed to reflect AUC, such that lower AUC indicates greater sensitivity to drug treatment and a better response. The augmented PBNs were then trained by minimizing the normalized AUC values and derived phenotype scores in model-simulated steady-states across conditions defined by mutation status and drug inhibition. Figure 3C shows model fitness with test data after optimization, with a Pearson’s r of approximately 0.99 in the training set and 0.66–0.86 in the test set across all three drugs significantly. Results for other drugs can be found in Fig. S3. PBNs trained with different datasets featured with unique sets of parameters representing the probability of rules for each node.

The optimized PBNs recapitulate canonical *FLT3* signaling while revealing drug-specific rewiring around *FLT3* and apoptosis (Figure 3B; Fig. S4). Across all three drug-trained PBN, *FLT3* connects to core downstream effectors in the JAK/STAT5, PI3K/AKT, and RAS/MAPK pathways, consistent with established biology (27). In the PBN trained with **Entospletinib** drug sensitivity data, *FLT3* is partially regulated through a *SYK*-dependent rule, aligning with reports that *HOXA9/MEIS1*-high AML exhibits SYK-driven programs and heightened sensitivity to SYK inhibition (28). By contrast, the PBN trained with **Ibrutinib** data assigns the highest probabilities to rules in which downstream nodes depend directly on *FLT3*, mirroring preclinical data that BTK inhibition selectively targets *FLT3* mutated AML by suppressing STAT5, AKT, and ERK signaling (29). PBNs trained with **Venetoclax** and **Entospletinib** data instead distribute control of apoptosis signaling across multiple inputs and more strongly couple *HOXA9* and *BCL2* to the *DNMT3A/NPM1* mutational background and *TP53* status.

We demonstrated how KGBN elucidates the underlying regulatory effects with simulated probability between regulators and drug targets, we then ask whether it can be used to distinguish efficacy across mutations for different drugs (Figure 3D). After training on a combined dataset of the three drugs and testing on a separate cohort, we further validate the model’s performance on an external dataset of drug-response DSS scores from the FPMTB, all of which show a robust, strong correlation between model-predicted apoptosis and experimental measurements of cell viability after treatment.

#### 3.2.2 Model augmentation on mutations

**Fig. 4.**
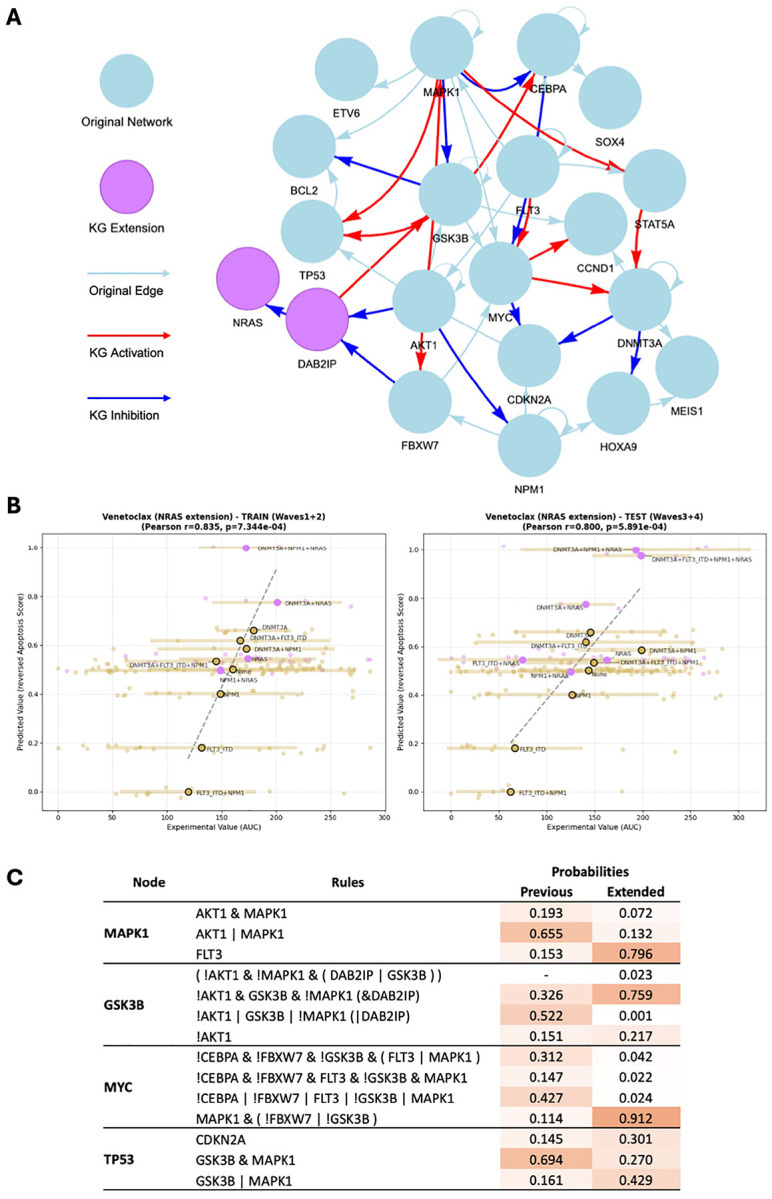
Augmentation of the Venetoclax model with NRAS mutation. (A) The extended model. (B) Correlation of simulation and measurements in train and test set. (C) Rule probabilities comparison in NRAS-extended PBN vs previous PBN.

Finally, we asked whether KGBN can be further leveraged to augment the model with additional recurrent mutations and thereby refine mutation-specific drug response. Beyond the core AML driver genes considered above, the workflow enables systematic incorporation of new genes and mutation profiles without restructuring the base model. As a proof of concept, we further augmented the model in 3.2.1 for Venetoclax to include *NRAS* and its regulations (Figure 4A). By re-training the PBN on drug response grouped by *FLT3*-ITD, *NPM1*, *DNMT3A*, and *NRAS* mutation status, we obtained an optimized model that covers additional patient mutation profile (purple-colored nodes in Figure 4B). This extension preserves predictive performance while enabling interrogation of how additional oncogenic alterations reshape regulatory dependencies under treatment. Results for augmenting the model to *NRAS* for other drugs are summarized in Fig. S5.

To interpret *NRAS*-associated rewiring, we examined rules whose probabilities changed most after model extension (Figure 4C, Fig. S6). For ***MAPK1***, optimized rules emphasize that *MAPK1* is driven directly by *FLT3*, consistent with FLT3/RAS signaling feeding into the MEK–ERK cascade, a pathway whose activation is known to confer MCL1-mediated resistance to Venetoclax in AML (30). For ***GSK3B***, the high-probability rule in the extended model requires absence of *AKT1* and *MAPK1* together with *DAB2IP*, whereas a more permissive OR rule dominated the original model; this pattern mirrors the known inhibition of GSK-3*β* by Ras/PI3K–AKT and MEK/ERK signaling, which stabilizes the anti-apoptotic protein *MCL1* and promotes Venetoclax resistance (31). For ***MYC***, *NRAS* extension strongly favors a rule in which *MYC* depends on *MAPK1* activity and loss of its negative regulators *GSK3B/FBXW7*, in line with Ras/ERK-mediated phosphorylation that stabilizes c-Myc and enhances leukemic self-renewal (32). Finally, ***TP53*** shifts from a strict condition toward more permissive inputs, consistent with knowledge about how oncogenic Ras activates the p53 stress-response pathway (33). Together with clinical evidence that RAS-pathway activation is associated with poor response to Venetoclax in AML (34), these rule-probability shifts support a model in which *NRAS* rewires the network toward Ras/MAPK- and MCL1-dependent survival pathways, thereby reducing leukemic cells’ reliance on *BCL2* (35).

## 4 Discussion

In this study, we introduced KGBN, a knowledge graph augmented framework to improve logical GRN models using probabilistic representations and experimental calibration. Applying the framework to AML, we demonstrated that augmenting an existing GRN with drug-target pathways and optimizing rule probabilities against *ex vivo* drug-response data allows the model to capture mutation-specific therapeutic sensitivities and decipher signaling dependencies.

A key strength of KGBN lies in its explicit representation of regulatory uncertainty. Rather than committing to a single deterministic logic, the PBN formulation allows multiple competing regulatory hypotheses to coexist, with their relative influence learned from data. The resulting drug-specific rule probability profiles revealed distinct regulatory rewiring patterns consistent with known AML biology, such as differential dependence on *FLT3* signaling across networks trained using Venetoclax, Entospletinib, and Ibrutinib data. This illustrates how data-trained PBNs can serve not only as predictive models but also as interpretable summaries of context-specific pathway utilization. Further, by grounding newly introduced nodes and edges in curated knowledge resources, KGBN provides transparent and traceable biological provenance for model augmentation. This is particularly valuable for modeling drug mechanisms of action and resistance, where regulatory context and pathway crosstalk play a critical role. Moreover, the phenotype scoring strategy enables alignment between qualitative network states and quantitative experimental readouts, bridging a longstanding gap between logical modeling and experimental data.

KGBN complements our prior work on LM-Merger, which integrates multiple existing GRN models to improve robustness and regulatory coverage (21). While LM-Merger demonstrated improved generalization by combining AML and hematopoiesis models (7; 36), KGBN addresses a complementary setting in which curated knowledge graphs are used to extend a single validated model with new genes, pathways, and regulatory hypotheses. Together, these approaches support flexible GRN construction through either model–model integration or knowledge-driven extension, depending on data availability and modeling goals.

Although this study focuses on AML as a primary use case, the workflow is not disease-specific. We have demonstrated its generalizability by reproducing a published pancreatic cancer logical model. Building on this validated baseline, we are currently augmenting the pancreatic cancer model with drug-target pathways and probabilistic logic to enable data-driven prediction of combination therapies. Beyond extending existing models, KGBN can use knowledge graphs to extract regulatory topology directly from a set of genes and phenotypes of interest and construct novel logical models for system-level simulation. More broadly, the modular design of KGBN allows integration of additional structured knowledge sources beyond SIGNOR. As a future direction, we are actively incorporating causal and drug-response knowledge from NCATS Translator resources to expand regulatory coverage, improve drug-target representation, and support cross-disease modeling (13). These extensions will further strengthen the workflow’s applicability to diverse contexts and advance its role as a general framework for GRN modeling.

We would like to acknowledge a few limitations of our study. First, the quality of model extension depends on the completeness and accuracy of underlying knowledge graphs, which may contain conflicting or context-agnostic interactions. While the probabilistic framework partially mitigates this issue, our future work will incorporate evidence weighting, diseasespecific filtering, or ensemble knowledge graph integration. Second, optimization against limited datasets risks overfitting, particularly for large networks. With the data we have, we have carried out cross-validation and external validation with another independent dataset. Another strategy to avoid overfitting would be to try model compression strategies, especially as models become larger.

Overall, KGBN advances logical GRN modeling by providing a reproducible, extensible, and data-informed pathway from curated mechanistic knowledge to predictive, context-aware models. By enabling systematic incorporation of drug targets, regulatory uncertainty, and experimental calibration, this framework lays the groundwork for scalable mechanistic modeling in systems biology and supports the broader vision of precision medicine.
